# PRX1 knockdown potentiates vitamin K3 toxicity in cancer cells: a potential new therapeutic perspective for an old drug

**DOI:** 10.1186/s13046-015-0270-2

**Published:** 2015-12-21

**Authors:** Tiantian He, Elie Hatem, Laurence Vernis, Ming Lei, Meng-Er Huang

**Affiliations:** Centre National de la Recherche Scientifique, UMR3348 “Genotoxic Stress and Cancer”, Centre Universitaire, Orsay, 91405 France; Institut Curie, Centre de Recherche, Orsay, 91405 France; Northwest A&F University, College of Life Science, Key Laboratory of Agricultural Molecular Biology, Yangling, Shaanxi Province 712100 China

**Keywords:** Peroxiredoxin 1, Vitamin K3, Menadione, Reactive oxygen species, Redox modulation, Drug sensitization

## Abstract

**Background:**

Many promising anticancer molecules are abandoned during the course from bench to bedside due to lack of clear-cut efficiency and/or severe side effects. Vitamin K3 (vitK3) is a synthetic naphthoquinone exhibiting significant in vitro and in vivo anticancer activity against multiple human cancers, and has therapeutic potential when combined with other anticancer molecules. The major mechanism for the anticancer activity of vitK3 is the generation of cytotoxic reactive oxygen species (ROS). We thus reasoned that a rational redox modulation of cancer cells could enhance vitK3 anticancer efficiency.

**Methods:**

Cancer cell lines with peroxiredoxin 1 (PRX1) gene transiently or stably knocked-down and corresponding controls were exposed to vitK3 as well as a set of anticancer molecules, including vinblastine, taxol, doxorubicin, daunorubicin, actinomycin D and 5-fluorouracil. Cytotoxic effects and cell death events were evaluated by 3-(4,5-dimethylthiazol-2-yl)-2,5-diphenyltetrazolium bromide (MTT)-based assay, cell clonogenic assay, measurement of mitochondrial membrane potential and annexin V/propidium iodide double staining. Global ROS accumulation and compartment-specific H_2_O_2_ generation were determined respectively by a redox-sensitive chemical probe and H_2_O_2_-sensitive sensor HyPer. Oxidation of endogenous antioxidant proteins including TRX1, TRX2 and PRX3 was monitored by redox western blot.

**Results:**

We observed that the PRX1 knockdown in HeLa and A549 cells conferred enhanced sensitivity to vitK3, reducing substantially the necessary doses to kill cancer cells. The same conditions (combination of vitK3 and PRX1 knockdown) caused little cytotoxicity in non-cancerous cells, suggesting a cancer-cell-selective property. Increased ROS accumulation had a crucial role in vitK3-induced cell death in PRX1 knockdown cells. The use of H_2_O_2_-specific sensors HyPer revealed that vitK3 lead to immediate accumulation of H_2_O_2_ in the cytosol, nucleus, and mitochondrial matrix. PRX1 silencing significantly up-regulated mRNA and protein levels of NRH:quinone oxidoreductase 2, which was partially responsible for vitK3-induced ROS accumulation and consequent cell death.

**Conclusion:**

Our data suggest that PRX1 inactivation could represent an interesting strategy to enhance cancer cell sensitivity to vitK3, providing a potential new therapeutic perspective for this old molecule. Conceptually, a combination of drugs that modulate intracellular redox states and drugs that operate through the generation of ROS could be a new therapeutic strategy for cancer treatment.

## Background

Vitamin K3 (2-methyl-1, 4 naphthoquinone, also known as menadione) is a form of vitamin K that does not participate in the synthesis of coagulation proteins [1, 2]. Rather, vitamin K3 (vitK3) is readily redox cycled, thereby generating reactive oxygen species (ROS) and consuming NADPH. VitK3 exhibits anticancer activity against a variety of human cancer cell lines [3–6]. The association of vitK3-induced cell death with the cellular depletion of glutathione, NADPH oxidation, the occurrence of macromolecular damage, and the disruption of calcium homeostasis, support the notion that the anticancer activity of vitK3 is linked to oxidative stress [3, 7–9].

The encouraging results obtained in cell models regarding vitK3 anticancer activity have prompted several in vivo investigations. A clinical phase I study showed that vitK3 is reasonably well-tolerated, and the concentrations that are required for suppressing tumor growth in vitro are clinically achievable [3, 10]. On the basis of the synergistic cytotoxicity between vitK3 and other anticancer drugs such as mitomycin C, vitamin C, or radiation in vitro [2], clinical trials evaluating the therapeutic effect of these combinations have been carried out. An early study showed that vitK3 potentiates radiation efficiency in patients with buccal carcinoma [3], but a clinical phase II trial of vitK3 and mitomycin C combination in advanced lung cancer patients and gastrointestinal cancer patients failed to demonstrate benefit [2, 11]. However, a clinical phase I/IIa study of the combination of vitK3 and vitamin C in a group of end stage prostate cancer patients who failed standard therapy showed a strong synergistic effect [12]. Taken together, these mixed results indicate that the cancer types, doses of vitK3 and its combination with other drugs, influence the effectiveness of vitK3 as an adjuvant or co-adjuvant in chemotherapy. Additional studies are clearly warranted to optimize the anticancer therapeutic effect of vitK3 and to minimize its toxic side effects.

ROS generation is considered as a key mechanism of vitK3-induced cell death [2, 13], and ROS-mediated cancer cell killing is receiving increasing attention [14–16]. However, due to the presence of oxidative stress adaptive mechanisms, the use of ROS-generating agents alone may not be sufficient to efficiently kill cancer cells. Therefore, combinations of ROS-generating agents with compounds capable of abrogating cellular antioxidant defense systems are likely to have an additive or even synergistic effect. This approach might be particularly useful for the treatment of drugs-resistant cancer cells that have become adapted to stress. We thus reasoned that redox modulation of cancer cells through peroxiredoxin 1 (PRX1) knockdown could enhance the anticancer activity of vitK3. Peroxiredoxins constitute a large family of thiol-based peroxidases that reduce peroxides with electrons donated by thioredoxin 1 (TRX1) [17, 18], and PRX1 is the most abundant of the six human peroxiredoxins. Importantly, PRX1 is not only important for cellular peroxide scavenging, but also regulates numerous signaling pathways via direct protein-protein interaction with, for instance, ASK1, GSTpi-JNK, AR, PTEN and c-ABL [19–21], or by modulating H_2_O_2_ signaling [21]. PRX1 is frequently over-expressed in various cancer cells including lung, bladder, liver, thyroid, and breast cancers [22–24], and its over-expression has been associated with carcinogenesis, metastasis and resistance to radiotherapy or chemotherapy [22, 23, 25]. In the present study, we observed that PRX1 knockdown (transient or stable silencing) enhanced the toxic effect of vitK3 on cancer cells (A549 and HeLa) with little or no effect on non-transformed immortalized cells and primary fibroblasts. This selective cytotoxicity in PRX1 knockdown cancer cells is associated with a significant cellular accumulation of H_2_O_2_ partially due to an overexpression of NRH:quinone oxidoreductase 2 (NQO2). PRX1 inactivation could represent an interesting strategy to enhance cancer cell sensitivity to vitK3, providing a potential new therapeutic perspective for this old molecule.

## Methods

### Reagents

VitK3, N-ethylmaleimide (NEM), N-acetyl-L-cysteine (NAC), dimethyl sulfoxide (DMSO), dithiothreitol (DTT), H_2_O_2_ and trichloroacetic acid (TCA) were purchased from Sigma-Aldrich (St. Louis, MO, USA). Vinblastine, taxol, doxorubicin, daunorubicin, actinomycin D and 5-fluorouracil were from Enzo Life Sciences (Villeurbanne, France). Hygromycin B, 6-carboxy-2',7'-dichlorodihydrofluorescein diacetate (carboxy-H_2_DCFDA), 3-(4,5-dimethylthiazol-2-yl)-2,5-diphenyltetrazolium bromide (MTT), crystal violet, Vybrant Apoptosis Assay Kit #3, propidium iodide (PI), tetramethylrhodamine methyl ester (TMRM), 4-acetamido-4’-maleimidylstilbene-2,2’-disulfonic acid (AMS) were from Life Technologies (Eugene, OR, USA). The following antibodies were used: anti-PRX1 (AbFrontier, Seoul, Korea); anti-PRX3 (Abcam, Cambridge, MA, USA); anti-TRX2 (R&D Systems, Minneapolis, MN, USA); anti-NQO2 (Proteintech, Chicago, IL, USA); anti-GFP (Life Technologies); anti-β-actin (Santa Cruz Biotechnology); anti-α-tubulin (Sigma-Aldrich) and anti-TRX1 (Cell Signaling Technology, Danvers, MA, USA). ON-TARGETplus SMARTpool small interfering RNA (siRNA) targeting PRX1 (siPrx1), and non-targeting pool control siRNA (siCon) were from Dharmacon (Lafayette, CO, USA). Mammalian expression vectors encoding HyPer targeted to cytosol, nucleus and mitochondria were purchased from Evrogen (Moscow, Russia).

### Cell culture

Human cervical adenocarcinoma HeLa and human lung cancer A549 cell lines were from American Type Culture Collection (ATCC, Manassas, VA, USA) and were cultured in standard Dulbecco’s modified Eagle’s medium (DMEM) with 10 % fetal bovine serum (FBS). Human vascular endothelial cell line HUVEC was from ATCC and cultured in standard DMEM with 10 % FBS. Primary normal dermal fibroblasts from ATCC were kindly provided by Dr. Matthieu Gratia (UMR3348 CNRS-Institut Curie, Orsay, France) and cultured in DMEM with 20 % FBS. The HeLa cells stably knocked-down for PRX1 gene (PRDX1 HeLa SilenciX, designated as Prx1– cells) and non-silenced control cells (Control HeLa SilenciX, designated as Prx1+ cells) were purchased from tebu-bio (Le Perray en Yvelines, France) and were cultured in DMEM supplemented with 50 μg/mL hygromycin B and 10 % FBS. All cell lines have been characterized and the last cell line authentication has been performed by eurofins (Ebersberg, Germany) using PCR-single-locus technology after finalization of the present study.

### siRNA and plasmid transfection

For siRNA transfection, HeLa, A549 and HUVEC cells and primary normal dermal fibroblasts were seeded at density of 4 × 10^3^ per well into a 96-well plate or at 1 × 10^5^ per well into a 6-well plate the day before transfection. Cells were then transfected with 5 nM siRNA (for HeLa, A549 and HUVEC cells) or 15 nM siRNA (for primary normal dermal fibroblasts) using INTERFERin reagents (Polyplus Transfection, Illkirch, France) according to the manufacturer’s instructions. Further experiments were performed 48 h after transfection. For HyPer transfection, HeLa cells were seeded at density of 4 × 10^5^ per well into a 6-well plate the day before transfection and were then transfected with 1 μg of HyPer expressing vectors using jetPRIME reagents (Polyplus Transfection).

### Cell viability and cell death assays

MTT-based cell viability assay was performed as previously reported [26]. For cell clonogenic assay, ~300 cells per well were plated in 6-well plates and cultured at 37 °C overnight. Cells were then treated with indicated doses of vitK3 or DMSO (as vehicle control) for defined times followed by incubation in fresh medium for another 10 days. The culture medium was changed with fresh medium every 3 days. The cells were then fixed and stained with 0.05 % crystal violet in 70 % ethanol. The number of visible colonies was counted manually. Mitochondrial membrane potential (MMP) was monitored by TMRM staining, for which cells were washed with PBS and incubated with 10 nM TMRM for 30 min at 37 °C before being analyzed by flow cytometry. Fluorescent isothiocyanate (FITC)-conjugated annexin V/PI double staining was performed using the Vybrant Apoptosis Assay Kit #3 according to the manufacturer’s instructions. Cells were analyzed in FL1-H (FITC) and FL2-H (PI) channels on flow cytometer equipped with CellQuest software (FACSCalibur, Becton Dickinson).

### Measurement of ROS

The general intracellular ROS was assessed using carboxy-H_2_DCFDA. Briefly, cells were trypsinized, resuspended in medium without phenol red and serum, and stained with 10 μM carboxy-H_2_DCFDA for 30 min at 37 °C in the dark. The cells were then washed in PBS, collected and analyzed immediately by flow cytometry.

### Microscopy imaging

Real-time imaging of the H_2_O_2_ dynamics was monitored with the genetically encoded fluorescent probe HyPer [27]. Fluorescent imaging in living cells was performed using the microscope Leica DMI6000 B equipped with Leica MM AF Imaging System. Images were acquired using excitation filters YFPex (490–510 nm) and CFPex (420–445 nm) and emission filter YFPem. Time-lapse imaging was recorded every 10 s with Plan Fluotar dry objective (magnification 40×, numerical aperture 0.6). Images were then subtracted from background and filtered with median method in ImageJ software (National Institutes of Health, Bethesda, MD, USA). Ratio from average fluorescence intensity of channel YFPex/YFPem (F) divided by that of channel CFPex/YFPem (F0) was calculated frame by frame, followed by normalization to the value of images at start point of vitK3 treatment.

### Western blotting and redox western blotting

For classic western blotting, cells were lysed in M-PER protein extraction reagent (Pierce, Rockford, IL, USA) supplemented with 0.5 mM DTT and complete protease inhibitor cocktail (Roche Diagnostics, Mannheim, Germany). Lysates were resolved by 4–12 % (for PAR detection) or 12 % NuPAGE Bis-Tris gel (for other proteins) with MOPS buffer (Life Technologies) under reducing condition. Redox western blotting method was previously described [26]. Briefly, cells in culture were acid-quenched with ice-cold 10 % TCA, harvested, washed with acetone and dissolved in lysis buffer (100 mM Tris-Cl, 1 % SDS, 10 mM EDTA, pH 8.8) supplemented with 30 mM AMS (for TRX1 and TRX2) or 25 mM NEM (for PRX3 and HyPer). Following 2 h incubation under shaking at 37 °C (for AMS-treated samples) or 30 °C (for NEM-treated samples), insoluble protein was removed by centrifugation. Each sample containing about 30 μg of protein was separated by 4–12 % Bis-Tris gel with MOPS buffer (for HyPer) or 12 % Bis-Tris gel with MES buffer (for TRX1, TRX2 and PRX3, Life Technologies) under non-reducing condition. For both classic and redox western blotting, blots after incubation with first antibodies were washed and probed with IRDye 680- or IRDye 800cw-conjugated secondary antibody (LI-COR Biosciences, Lincoln, NE, USA) and then analyzed using an Odyssey Infrared Imaging System (LI-COR). Scanned images and band integrated intensity were analyzed and quantified by Odyssey v3.0 software.

### Quantitative real-time PCR (qRT-PCR)

Total RNA was isolated with RNeasy Protect Mini Kit (Qiagen GmbH, Hilden, Germany). cDNA was generated using iScript cDNA Synthesis Kit (Bio-Rad, Hercules, CA, USA). Forward and reverse primers for GAPDH and NQO2 genes were synthesized according to the sequence previously described [28, 29]. GAPDH served as a reference gene for relative quantification. qRT-PCR was performed with iQ SYBR Green Supermix (Bio-Rad) in a CFX96 real-time PCR detection system (Bio-Rad).

### Statistical analysis

All the values are the means ± SD of not less than three measurements except indicated. Comparisons between groups were analyzed using Student’s *t*-test. *P* values of ≤0.05 were considered significant.

## Results

### PRX1 knockdown confers selective sensitivity to vitk3 in cancer cell lines

**Fig. 1:**
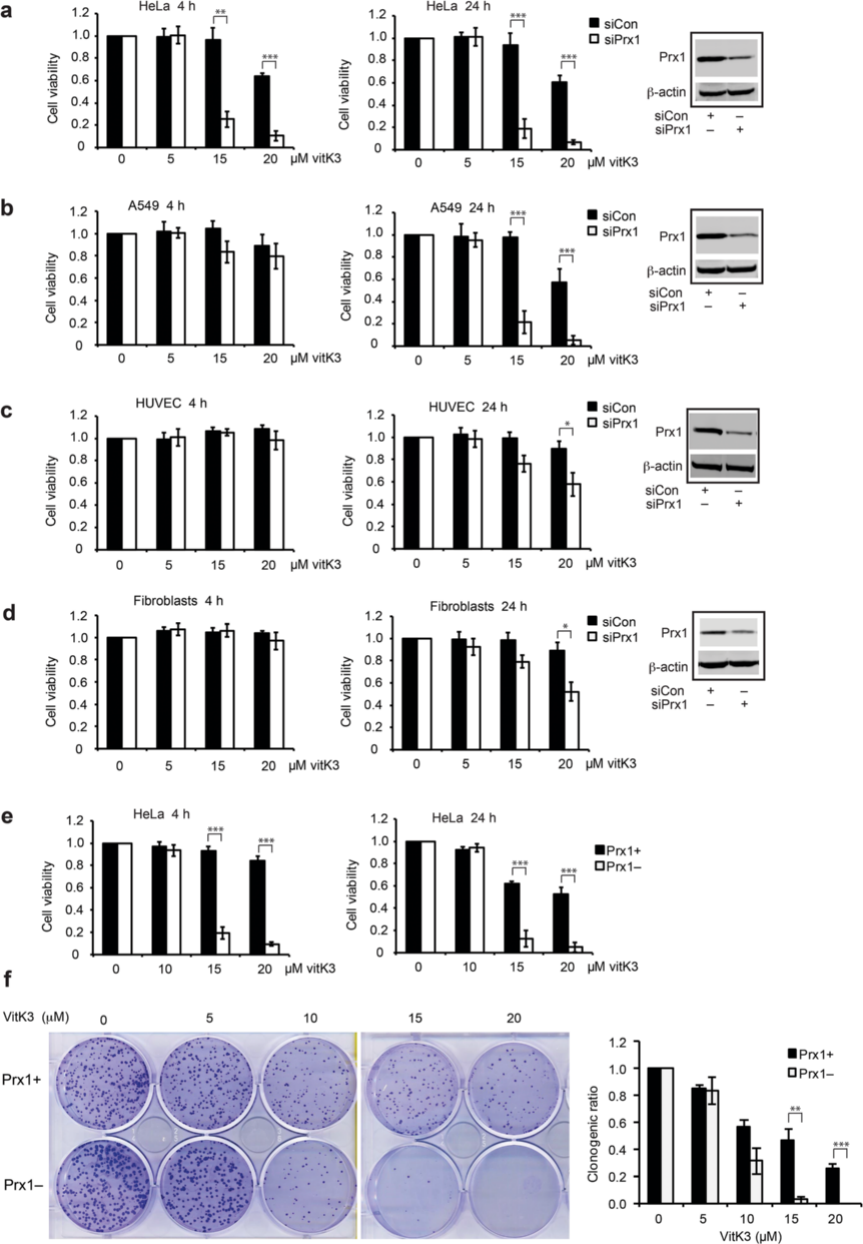
PRX1 silencing enhances cancer cell sensitivity to vitK3. **a**–**d** MTT assay on HeLa (**a**), A549 (**b**), HUVEC (**c**) cells and primary fibroblasts (**d**) transiently transfected with PRX1 specific siRNA or control siRNA for 48 h followed by vitK3 treatment with indicated doses for 4 and 24 h. The inserts are western blot to verify efficiency of PRX1 knockdown in siRNA transfected cells. **e** MTT assay on HeLa cells with stable PRX1 knockdown (Prx1–) and non-silenced control cells (Prx1+) treated with indicated doses of vitK3 for 4 and 24 h. **f** Clonogenic assay for Prx1+ and Prx1– cells treated with indicated doses of vitK3 for 4 h then recovered for 10 days. Representative scanned images of 6-well plates are presented (left panel). Clonogenic ratio = (colony number in vitK3-treated sample / colony number in DMSO-treated control sample) (right panel). Data are mean ± SD of at least three independent experiments. *P ≤ 0.05, **P ≤ 0.01, ***P ≤ 0.001

HeLa and A549 cancer cells were used to evaluate the role of PRX1 in cancer cell resistance to vitK3. PRX1 was transiently depleted with specific siRNA (siPrx1) and the reduction of PRX1 protein levels was validated by western blot (Fig. 1a and b). Based on the MTT assay, vitK3 was significantly more efficient at inducing cell death in PRX1-silenced cells than in control cells transfected with non-targeting pool control siRNA (siCon). After 4 h of 15 μM vitK3 treatment, viability of PRX1 knockdown HeLa cells was reduced to ~25 %, significantly lower than the control cells transfected with siCon (Fig. 1a). PRX1 knockdown A549 cells were more resistant (~80 % cell viability) than PRX1 knockdown HeLa cells (Fig. 1a, b). However, a 24 h treatment with 15 μM vitK3 caused the death of ~80 % of both PRX1-silenced HeLa and A549 cells compared with control cells that had a high survival rate (only ~10 % of cell death). In order to determine the vitK3 cytotoxicity in non-cancerous cells, siPrx1 or siCon transfected HUVEC and primary fibroblasts were exposed to different concentrations of vitK3 for 4 or 24 h. As shown in Fig. 1c and d, both PRX1-silenced HUVEC and fibroblast cells showed significantly higher resistant to 15 μM vitK3 treatment compared with PRX1-silenced HeLa and A549 cells. Interestingly, PRX1 knockdown did not potentiate vitK3 cytotoxic effect in HUVEC and fibroblast cells as cell viability was only reduced by ~20 %. This indicates that PRX1 knockdown confers vitK3 a cancer-cell-selective cytotoxicity which could be linked with the intrinsic property of malignant transformation.

In order to decipher the mechanisms underlying cancer-cell-selective cytotoxicity of vitK3 in PRX1-silenced cancer cells, we decided to use a cancer cell model where PRX1 was stably knockdown. To this end, we employed commercially available HeLa (Prx1–) and the corresponding control (Prx1+) cell lines. The efficiency of PRX1 knockdown was previously confirmed by qRT-PCR and western blot [26]. Based on the MTT assay, Prx1– cells exhibited a significant and highly reproducible hypersensitivity to vitK3. Similar to transient knockdown experiments, 4 h treatment with 15 μM vitK3 killed ~80 % of Prx1– cells while Prx1+ cells were not or only barely affected; after a 24 h treatment, ~90 % of Prx1– cells were dead, versus ~40 % for Prx1+ cells (Fig. 1e). Clonogenic assay further confirmed the enhanced vitK3 cytotoxicity in Prx1– cells. The clonogenic ratio of Prx1– cells was <5 % versus ~50 % for Prx1+ cells upon exposure to 15 μM vitK3 for 4 h (Fig. 1f).

**Fig. 2:**
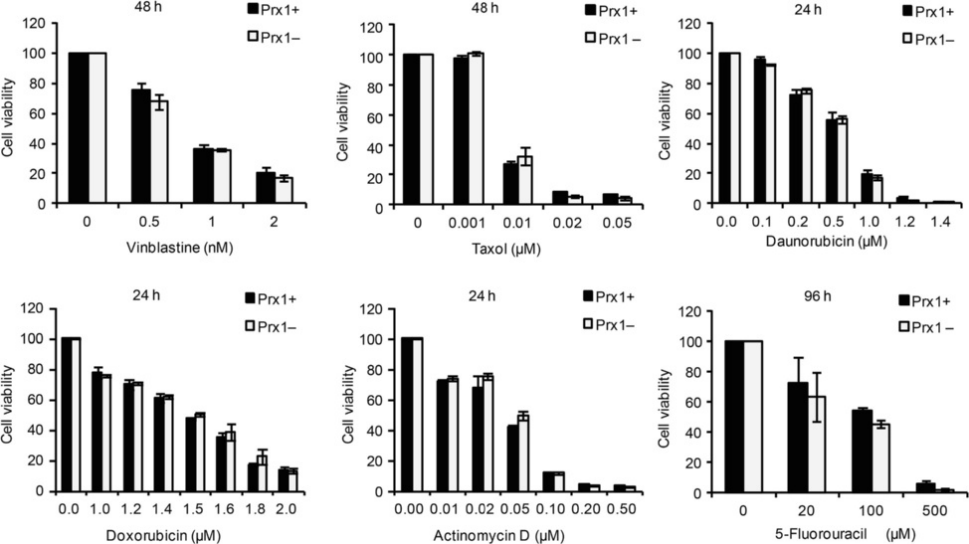
Cell viability of Prx1+ and Prx1– cells in response to different anti-cancer drugs. Both Prx1+ and Prx1– cells were treated with different doses of indicated drugs for defined times. Cell viabilities were monitored by MTT cell viability assay. Data are mean ± SD of at least 2 independent experiments

To determine whether the potentiating effect of PRX1 knockdown was specific to vitK3, we compared the cytotoxic effect of six known anticancer agents, vinblastine, taxol, doxorubicin, daunorubicin, actinomycin D, and 5-fluorouracil, on Prx1+ cells and Prx1– cells. These drugs have different mechanisms of action: vinblastine and taxol are microtubule disrupting agents; doxorubicin and daunorubicin are DNA intercalating agents; actinomycin D inhibits DNA transcription; and 5-fluorouracil arrests cell cycle [30]. Prx1+ and Prx1– cells exhibited a similar sensitivity to each of these drugs (Fig. 2). We conclude that both transient and stable PRX1 knockdown specifically potentiates vitK3 cytotoxicity on cancer cell lines employed in this study. This effect might be due to redox modulation, as vitK3 is the only ROS-generating agent among the seven drugs tested.

### The enhanced sensitivity to vitK3 involves possibly necroptosis

**Fig. 3:**
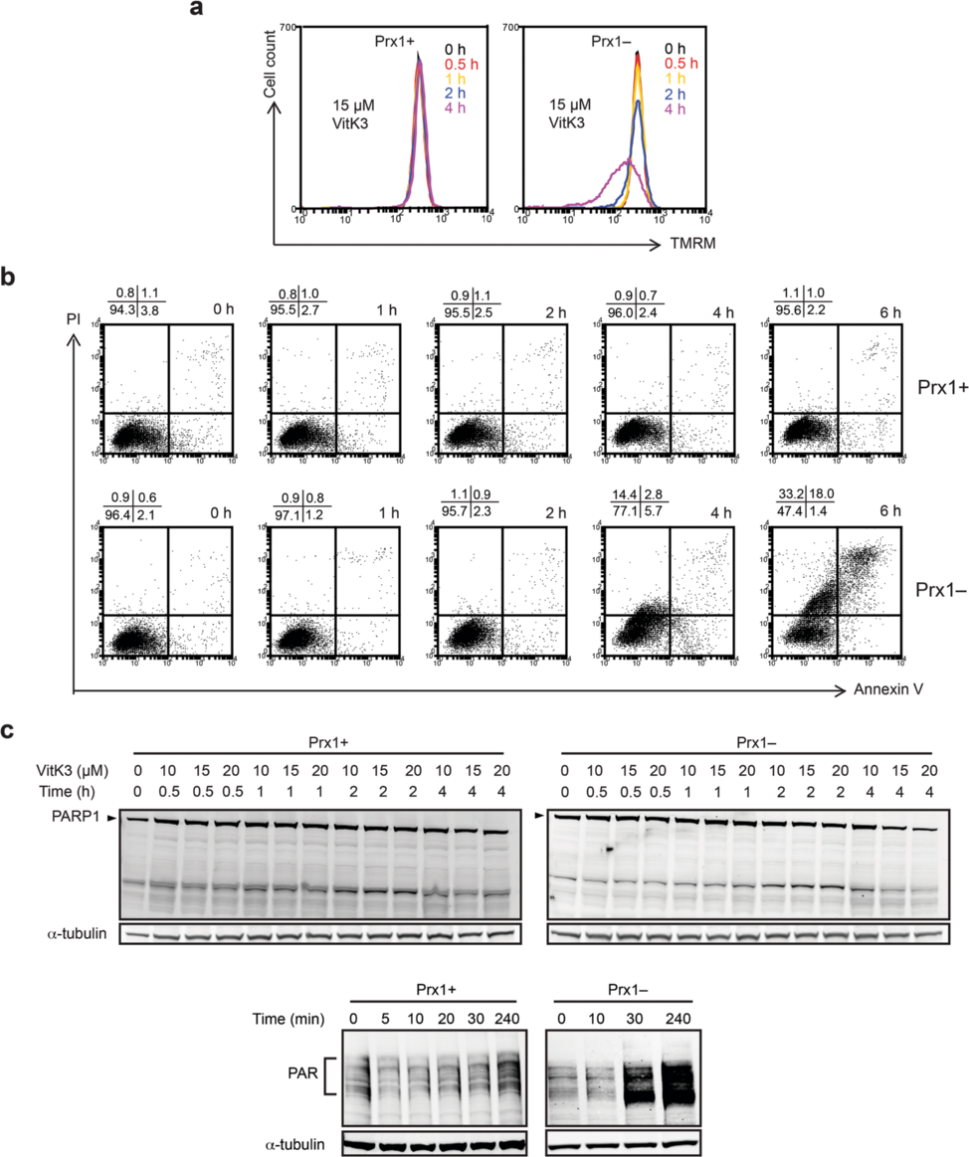
VitK3-induced cytotoxicity in Prx1– cells involves necroptosis-like death. **a** Detection of MMP by TMRM in cells exposed to vitK3 at indicated time points. One set of three independent results is presented. **b** Time course of annexin V/PI double staining in cells exposed to 15 μM vitK3. One representative set of dot plots is presented to show distribution of different cell populations at indicated time points. Percentage of each population is indicated. **c** Detection of PARP1 cleavage and PAR activation by western blot in the presence of indicated concentrations of vitK3 for defined times

VitK3 has been reported to induce both apoptosis [31] and necrosis [32]. We therefore assessed the nature of the cell death caused by vitK3 in Prx1– cells. TMRM staining allows monitoring the loss of MMP, which is a key early event in the process of apoptosis or programmed necrosis [33, 34]. The presence of 15 μM vitK3 during 4 h led to the loss of MMP that was seen in a significant proportion of Prx1– cells (~50 %), but not in Prx1+ cells (Fig. 3a). Similarly, a 6 h treatment with 15 μM vitK3 led to the appearance of annexin V^+^/PI^+^ cells in high proportion (>50 %) in the Prx1– but not Prx1+ cells (Fig. 3b). Taken together, both indicators suggest a rapid induction of cell death by vitK3 in Prx1– cells. Time course analysis showed that Prx1– cells underwent a clear shift from annexin V^–^/PI^–^ to both annexin V^–^/PI^+^ and annexin V^+^/PI^+^ cells without cells displaying annexin V^+^/PI^–^ staining (Fig. 3b), which suggests the occurrence of a programmed necrosis-like cell death, often termed necroptosis [35]. This observation was confirmed by the absence of vitK3-induced caspase-3 cleavage, an important executor in both extrinsic and intrinsic caspase-dependent apoptotic pathways (data not shown). Although Prx1+ and Prx1– cells both did not display any significant cleavage of poly (ADP-ribose) polymerase 1 (PARP1) during vitK3 treatment, we detected a significant increase in protein poly-ADP-ribosylation in Prx1– cells treated with vitK3 for 30 min (Fig. 3c). This latter result suggests the presence of an hyperactivation of PARP1 and further supports the notion that necroptosis constitute the mechanism of vitK3-induced Prx1– cell death [35].

### A major accumulation of H_2_O_2_ underlies Prx1– cells sensitivity to vitK3

**Fig. 4:**
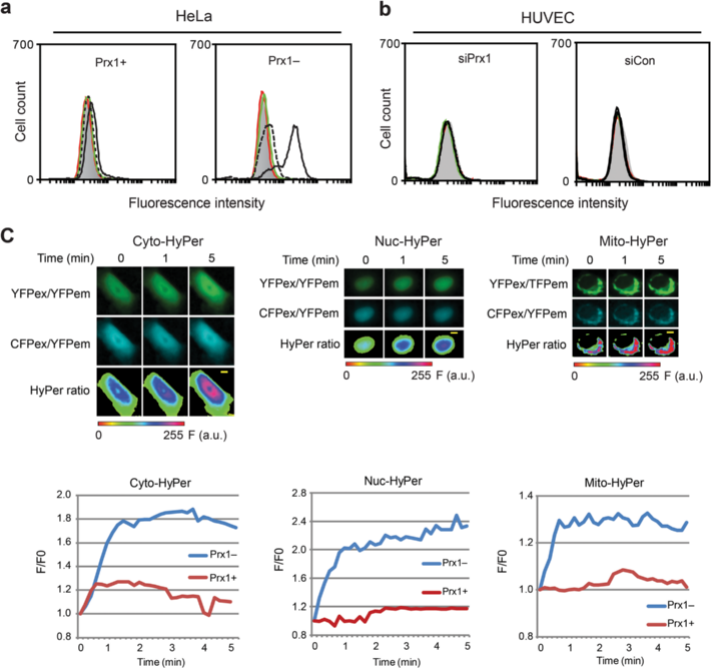
VitK3-induced ROS accumulation in Prx1– cells. **a**–**b** Measurement of intracellular ROS level using carboxy-H_2_DCFDA in Prx1+ and Prx1– HeLa cells (**a**) and in siPrx1- and siCon-treated HUVEC cells (**b**) after 15 μM vitK3 treatment. Results are plotted as cell counts versus fluorescence intensity. Gray shade, red line, green line, black dotted line and black solid line indicate the fluorescence intensity of the cells before treatment and after vitK3 treatment for 0.5, 1, 2 and 4 h, respectively. One set of three independent experiments is presented. **c** Fluorescence microscope and image analysis of cells expressing cyto-, nuc- and mito-HyPer. The time point before the addition of vitK3 is set as “0”. At upper panel, the upper and middle rows show acquired images under the YFPex/YFPem and the CFPex/YFPem channels. The lower row of images represents distribution of HyPer ratio. Scale bar = 6 μm. The color scale for the ratio values represents arbitrary unit of the spectrum, with less oxidized HyPer in green and highly oxidized HyPer in peak red. At lower panel, time course for the F/F0 ratio values (see Materials and Methods) in single cell expressing cyto-, nuc- or mito-HyPer during vitK3 treatment is presented. Fluorescence intensity of HyPer was recorded every 10 s over 5 min

H_2_O_2_ generation is thought to be a key causative event in vitK3-induced cell death [13, 36], and the effect of the PRX1 knockdown might therefore operate through an increase in cellular H_2_O_2_ levels. We first used redox-sensitive chemical probe carboxy-H_2_DCFDA to evaluate general cellular ROS levels during vitK3 treatment. As show in Fig. 4a, treatment of 15 μM vitK3 for 4 h led to a strong increase in DCF fluorescence in Prx1– cells, but not in Prx1+ cells. In contrast, same treatment did not trigger visible change in DCF fluorescence in HUVEC cells (Fig. 4b) and in primary fibroblasts (data not shown) with or without PRX1 knockdown. Therefore, different levels of ROS accumulation in these cells in response to vitK3 are correlated with their different degrees of sensitivity to vitK3.

**Fig. 5:**
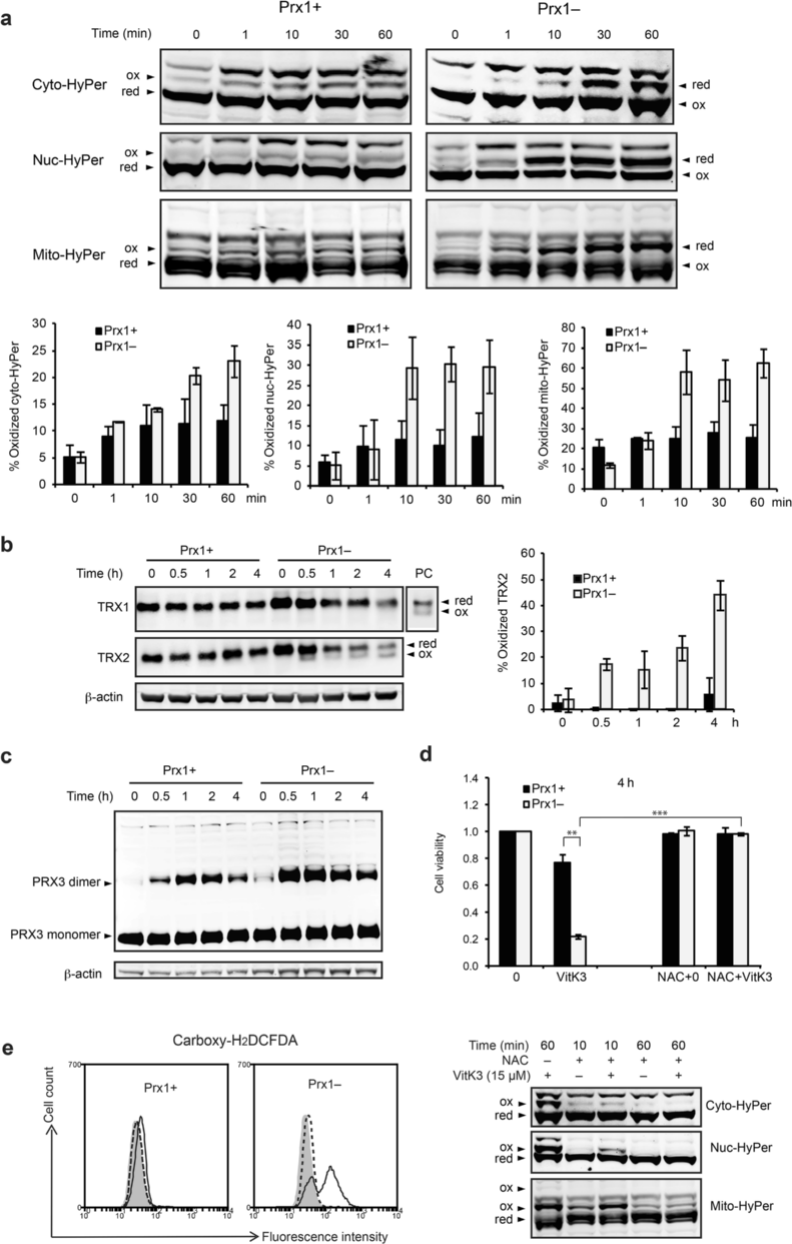
Oxidation of HyPer, endogenous TRX1, TRX2 and PRX3 monitored by redox western blot. **a** Oxidation of HyPer in response to vitK3 treatment. Cells expressing respectively cyto-, nuc- and mito-HyPer were treated with 15 μM vitK3 for indicated times and then subjected to redox western blots. Quantifications are mean ± SD of at least three independent experiments. **b** Redox western blot for TRX1 and TRX2. Cells treated with 15 μM vitK3 for indicated times were subjected to redox protein extraction and AMS treatment. Lane PC presents TRX1 oxidation in H_2_O_2_-treated Prx1+ cells as positive control. Quantifications are mean ± SD of at least three independent experiments. **c** Redox western blot for PRX3. Redox protein extraction was treated with NEM. Monomer and dimer forms are indicated. **d** NAC suppresses vitK3 toxicity. Cells were pretreated with 10 mM NAC for 2 h prior to incubation with freshly prepared medium containing both 10 mM NAC and 15 μM vitK3 for additional 4 h. Cell viability was determined by MTT assay (upper panel). **e** NAC suppresses vitK3-induced ROS accumulation. Global ROS levels were monitored by carboxy-H_2_DCFDA (left panel). Gray shade, black solid line, and black dotted line indicate the fluorescence intensity of the control cells, cells treated with 15 μM vitK3 alone for 4 h, and cells treated with 10 mM NAC and 15 μM vitK3 for 4 h, respectively. Compartment-specific H_2_O_2_ levels in Prx1– cells were monitored by HyPer probes (right panel). Redox state of HyPer in Prx1– cells treated with 15 μM vitK3 for 60 min was used as positive control. *P ≤ 0.05, **P ≤ 0.01, ***P ≤ 0.001

To get insights into the dynamics of H_2_O_2_ accumulation and its cellular compartmentation following vitK3 treatment, we used variants of the H_2_O_2_-specific sensor HyPer targeted to cytosol, nucleus or mitochondrial matrix (designated cyto-, nuc- and mito-HyPer) [27]. Fluorescence intensity of HyPer was recorded every 10 s upon vitK3 treatment. In Prx1– cells all three compartments displayed rapid and significant increase in fluorescence intensity, detectable 1 min after addition of vitK3 (Fig. 4c), while in Prx1+ cells no such increase was seen. We also recorded the time course oxidation of HyPer over 60 min in the presence of 15 μM vitK3 by separating the reduced versus oxidized forms of the probe by redox western blot (Fig. 5a). In Prx1– cells, addition of vitK3 caused a gradual oxidation/reduction of the HyPer over time in the three compartments, which started at 1 min and plateaued after about 10 min in the nucleus and mitochondria and at 20 min in the cytosol. It is worth noting that the oxidation of HyPer probe was much higher in mitochondria (~60 % at plateau) than in the cytosol (~25 % at 60 min) and nucleus (~30 % at plateau). In contrast, in each of the three compartments of Prx1+ cells exposed to vitK3, oxidation of HyPer was never over half of the oxidation seen in Prx1– cells. Both fluorescence microscopy and redox western blots suggest that vitK3 induces a rapid accumulation of H_2_O_2_ in all three compartments inspected, and that this accumulation is significantly higher in Prx1– cells, particularly in the mitochondrial matrix.

We next investigated the effect of the H_2_O_2_ produced upon vitK3 treatment on the expression and redox states of the cytosolic (TRX1) and mitochondrial (TRX2) thioredoxins and of PRX3 which is a peroxiredoxin located in the mitochondrial matrix and a major H_2_O_2_-scavenging enzyme within this compartment [37, 38]. As shown in Fig. 5b, TRX1 and TRX2 were mostly in the reduced form in both Prx1+ and Prx1– cells, TRX1 remained reduced upon addition of vitK3 while TRX2 became oxidized up to 50 % in Prx1– cells. On the other hand, TRX1 and TRX2 protein levels, which were initially elevated in Prx1– cells compared with Prx1+ cells, decreased by 2–3 fold upon drug treatment whereas they remained constant in Prx1+ cells (Fig. 5b). PRX3 levels were not different between Prx1– and Prx1+ cells; however the enzyme underwent rapid oxidation upon drug treatment, as manifested by the apparition of characteristic disulfide-linked dimers, and this oxidation was more pronounced in Prx1– cells (Fig. 5c). In summary, vitK3 treatment leads to pronounced redox alterations in Prx1– cells, including H_2_O_2_ accumulation, TRX2 and PRX3 oxidation, and a decrease in the protein levels of both TRX1 and TRX2. All may contribute to an enhanced vitK3 cytotoxicity.

To assess the causative role of ROS in vitK3-induced cell death, we tested the effect of the antioxidant NAC on cell survival. The addition of 10 mM NAC nearly completely prevented vitK3-induced death of Prx1– cells (Fig. 5d). NAC also prevented the accumulation of ROS and more specifically H_2_O_2_ after vitK3 treatment, as revealed by carboxy-H_2_DCFDA fluorescence and by the redox state of HyPer probes (Fig. 5e). These data strongly suggest that the increased ROS accumulation in Prx1– cells has a causative role in vitK3-induced cell death.

### Enhanced sensitivity to vitK3 in Prx1– cells relies on NQO2 expression

**Fig. 6:**
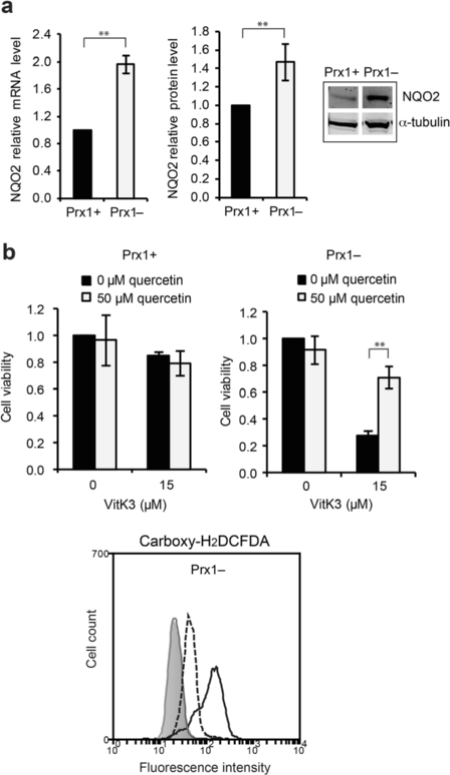
Increased expression of NQO2 in Prx1– cells accounts for enhanced cell death in response to vitK3 treatment. **a** NQO2 mRNA and protein levels were detected by qRT-PCR and western blot, respectively. The inserts are western blot detecting NQO2. The cropped blot images for NQO2 and loading controls are from same western blot. Quantifications are mean ± SD of three independent experiments. **b** Inhibition of NQO2 activity attenuated vitK3 toxicity. Cells pretreated with quercetin (NQO2 inhibitor) were exposed to a combination of quercetin and vitK3. Cell viability was determined by MTT assay. Data are mean ± SD of at least three independent experiments. Global ROS levels were monitored by carboxy-H_2_DCFDA staining. Gray shade, gray solid line, black solid line, and black dotted line indicate the fluorescence intensity of the control cells, cells treated with quercetin alone for 4 h, cells treated with vitK3 alone for 4 h, and cells treated with quercetin and vitK3 for 4 h, respectively. **P ≤ 0.01

Previous studies have demonstrated that NQO2 activity, which reduces vitK3 to its hydroquinone form without the formation of a semiquinone radical intermediate, is required for vitK3 redox cycling and cytotoxicity [1]. qRT-PCR and western blot analyses revealed that mRNA and protein levels of NQO2 were significantly up-regulated in Prx1– cells compared with control cells (Fig. 6a). To test whether the higher NQO2 levels in Prx1– cells could account for the enhanced toxicity of vitK3 in these cells, the NQO2 inhibitor quercetin [39, 40] was added prior to vitK3 treatment. Quercetin significantly attenuated vitK3-induced cell death and vitK3-induced ROS accumulation (Fig. 6b). These data confirm the implication of NQO2 in vitK3-induced cytotoxicity.

## Discussion

Rational drug combinations directed against multiple targets are effective anticancer strategies, by preventing cancer drug resistance and allowing the use of lower therapeutic drug posologies, thus overcoming the side effects of high doses of the single drugs. In the present study, we showed that PRX1 knockdown significantly and specifically potentiates vitK3 cellular sensitivity, which allows the reduction of the doses necessary to kill cancer cells. MTT and clonogenic assays, TMRM and annexin V/PI double staining all demonstrated that a 24 h treatment with 15 μM vitK3 is enough to trigger cell death and kill a significant proportion of PRX1 knockdown cancer cells. Importantly, such treatments caused little apparent reduction in cell viability of non-cancerous cells, with or without PRX1 knockdown. This indicates that vitK3 has a cancer-cell-selective cytotoxic property, in particular when PRX1 activity is compromised.

Using recently developed redox analysis tools, we demonstrated that 15 μM vitK3 triggered a rapid and significant accumulation of H_2_O_2_ in the mitochondrial matrix, cytosol and nucleus in Prx1– cells. This ROS accumulation of H_2_O_2_ played a determinant role in enhanced vitK3-induced cell death. Up-regulation of NQO2 in Prx1– cells appears to contribute to the increased H_2_O_2_ generation in the presence of vitK3. How PRX1 knockdown leads to the up-regulation of NQO2 remains unknown. NQO2 gene contains the antioxidant response element (ARE) in its promotor region and is part of the Nrf2-regulated defensive mechanism [41]. It is possible that PRX1 knockdown causes redox imbalance that up-regulates the expression of some Nrf2-regulated ARE-containing genes including NQO2. NQO2 catalyzes metabolic activation of vitK3 leading to cytotoxicity, in contrast to NADPH:quinone oxidoreductase (NQO1) that metabolically detoxifies vitK3 and protects cells against oxidative stress [1]. As 15 μM vitK3 induced only minor ROS accumulation and cytotoxicity in Prx1+ cells, we propose that the rapid and important ROS elevation in Prx1– cells could be derived from a combined effect of an increased H_2_O_2_ generation during vitK3 metabolism by higher NQO2 activity and a decreased PRX1 scavenging activity. Enhanced H_2_O_2_ accumulation in Prx1– cells upon vitK3 treatment is detected in the three subcellular compartments analyzed with a marked effect on mitochondrion. This was confirmed by the oxidation of TRX2 and PRX3, two major mitochondrial redox-maintaining-antioxidant proteins [42], which may reflect a widespread mitochondrial protein oxidation and is likely to further facilitate H_2_O_2_ accumulation in mitochondria, leading eventually to cell death.

VitK3 and β-lapachone (β-lap) both trigger remarkable accumulation of H_2_O_2_ in PRX1 knockdown HeLa cells [26]. However, although these two quinones are supposed to share similar redox cycling pathways, they implicate different mechanisms. ROS accumulation in vitK3-treated and β-lap-treated Prx1– cells depends on NQO2 and NQO1, respectively. In particular, NQO1 catalyzes a two-electron reduction of both vitK3 and β-lap [43]. While vitK3 is detoxified by NQO1 [44], β-lap toxicity is strongly potentiated by this enzyme activity [45]. On the other hand, inhibition of NQO2 (present study) or the absence of NQO2 (NQO2-null mice) confers resistance to vitK3-induced oxidative stress and cytotoxicity [1]. The role of NQO2 in β-lap toxicity remains unknown. Nonetheless, we observed that quercetin, a NQO2 inhibitor, significantly attenuated β-lap-induced cell death (data not shown), indicating the enhancing role of NQO2 in both β-lap and vitK3 cytotoxicity. In addition to distinct metabolisms related to ROS generation, the downstream events and signaling pathways that lead to cell death in response to β-lap and to vitK3 seems to be different. Among the MAPK pathways analyzed, JNK, p38 and ERK are highly phosphorylated after β-lap treatment and JNK activation plays a critical role in mediating low-dose β-lap-induced cell death in Prx1– cells [26]. However, an equivalent lethal dose of vitK3 results in moderate ERK phosphorylation that does not influence vitK3-induced Prx1– cell death (data not shown). It will be interesting to identify the distinct mechanisms of action of these two related quinone molecules, linking ROS accumulation and downstream signaling pathways.

VitK3-induced cancer-cell-selective toxicity is consistent with the notion that cancer cells usually display persistent pro-oxidative state. As such, cancer cells would be more dependent on the antioxidant system to maintain the ROS levels below the toxic threshold, and therefore more vulnerable to further oxidative stress induced by exogenous ROS-generating agents or by defective antioxidant system. Our current findings further support the view that modulation of intracellular redox states is a viable alternative approach to enhance cancer cell sensitivity to ROS-generating drugs, or to overcome some types of drug resistance [14, 15]. Elevated expression of PRX1 has frequently been found in various cancer cells [22, 24] and PRX1 is responsible for cell resistance to TRAIL and cisplatin [25, 46]. Therefore, PRX1 is an interesting target in an oxidative stress-modulating anticancer strategy. From this study, it is reasonable to expect a reduction of ~40 to ~50 % of vitK3 doses when administrated with an efficient PRX1 inhibitor, with better anti-cancer efficiency and less side effects. As no specific PRX1 inhibitor is currently commercially available, the reported peroxiredoxin inhibitors such as Conoidin A and Adenanthin inhibit preferentially peroxiredoxin 2 and affect other antioxidant pathways [47–49], the use of such inhibitors is less appealing for the establishment of a PRX1-based redox anticancer therapeutic strategy. Our data obtained by specific PRX1 knockdown approach provide a proof of concept that PRX1 could be an interesting anticancer target leading to a substantial reduction of the vitamin K3 dosage to kill cancer cells. The development of new specific and efficient PRX1 inhibitors could open new avenues for redox-based anticancer therapies.

## Conclusion

Killing cancer cells by a ROS-mediated mechanism has received increasing attention recently. However, due to the presence of redox adaptation mechanisms, the use of ROS-generating agents alone may not be sufficient to efficiently kill cancer cells exhibiting up-regulated antioxidants. Redox modulation of cancer cells by PRX1 knockdown confers enhanced sensitivity to vitK3 through increased ROS accumulation. PRX1 could be an anticancer target leading to a substantial reduction of the vitK3 dosage to kill cancer cells. Conceptually, a combination of drugs that modulate intracellular redox states and drugs that operate through the generation of ROS could be a new therapeutic strategy for cancer treatment.

## Abbreviations

AMS: 4-acetamido-4’-maleimidylstilbene-2,2’-disulfonic acid
carboxy-H_2_DCFDA: 6-carboxy-2',7'-dichlorodihydrofluorescein diacetate
DMEM: Dulbecco’s modified Eagle’s medium
DMSO: Dimethyl sulfoxide
DTT: Dithiothreitol
MMP: Mitochondrial membrane potential
FITC: Fluorescent isothiocyanate
H_2_O_2_: Hydrogen peroxide
β-lap: β-lapachone
MTT: 3-(4,5-dimethylthiazol-2-yl)-2,5-diphenyltetrazolium bromide
NAC: N-acetyl-L-cysteine
NEM: N-ethylmaleimide
NQO1: NADPH:quinone oxidoreductase
NQO2: NRH:quinone oxidoreductase 2
PI: Propidium iodide
qRT-PCR: Quantitative real-time PCR
ROS: Reactive oxygen species
PRX1: Peroxiredoxin 1
siRNA: Small interfering RNA
siPrx1: siRNA targeting PRX1
siCon: Non-targeting pool control siRNA
TCA: Trichloroacetic acid
TMRM: Tetramethylrhodamine methyl ester
TRX1: Thioredoxin 1
TRX2: Thioredoxin 2
vitK3: Vitamin K3
